# Psychosocial functioning in adolescents with non-suicidal self-injury: the roles of childhood maltreatment, borderline personality disorder and depression

**DOI:** 10.1186/s40479-021-00161-x

**Published:** 2021-07-01

**Authors:** Denisa Ghinea, Anna Fuchs, Peter Parzer, Julian Koenig, Franz Resch, Michael Kaess

**Affiliations:** 1 Department of Child and Adolescent Psychiatry, Centre for Psychosocial Medicine, University of Heidelberg, Heidelberg, Germany; 2 University Hospital of Child and Adolescent Psychiatry and Psychotherapy, University of Bern, Bern, Switzerland

**Keywords:** Childhood maltreatment, Depression, Borderline personality disorder, Psychosocial functioning, Structural equation model

## Abstract

**Background:**

There is a lack of studies examining psychosocial functioning in patients with non-suicidal self-injury (NSSI), especially in adolescents, and rates of impaired functioning in existing literature vary considerably. These variations may be attributable to further risk factors that influence psychosocial functioning. Thus, the aim of the study was to examine whether adolescent NSSI patients with childhood maltreatment (CM), a known risk factor for lower psychosocial functioning, may differ from adolescent NSSI patients without CM, and whether these differences may be explained by the severity of comorbid disorders. Specifically, we examined whether severity of borderline personality disorder (BPD), depression and posttraumatic stress disorder may explain differences in psychosocial functioning in NSSI patients with and without CM.

**Methods:**

Data of 368 adolescents with NSSI disorder from an outpatient clinic were analyzed using structural equation modeling. Clinicans’ rating of the *Global Assessment of Functioning Scale* (GAF) was collected, in addition to clinical interviews.

**Results:**

Results indicate that GAF scores were lower among NSSI patients with CM and that the difference in psychosocial functioning between these groups was explained by BPD and depression severity.

**Conclusions:**

Psychosocial functioning in NSSI patients varies depending on whether they have experienced CM or not. Specifically, these differences seem to be attributable to higher BPD and depression severity in adolescent NSSI patients with CM. Clinicians should ensure to assess CM and focus on BPD and depression severity in this population. Treatment of BPD and depression may notably reduce psychosocial impairment in NSSI patients with CM.

## Introduction

Psychosocial functioning describes a person’s ability to play their part in society by carrying out roles and performing activities in daily life such as self-preservation and basic living skills, work, school or leisure activities and interactions with their social environment [1, 2]. There is some indication that psychosocial functioning is significantly reduced in populations engaging in non-suicidal self-injury (NSSI) [3–5]. NSSI is defined as the direct, intentional destruction of one’s bodily tissue without the intent to die [6]. It is highly prevalent in adolescent populations with rates around 17–18% [7, 8] and rates up to 60% in child and adolescent clinical settings [9]. NSSI peaks around the age of 15 with remission in young to middle adulthood [10]. It is known to be associated to a wide range of psychiatric conditions [11, 12], nonetheless also occurring as an own diagnostic entity [5].

Studies that examine psychosocial functioning in NSSI populations are generally scarce, and existing work has mostly focused on adult populations [3, 4]. Rates of impaired psychosocial functioning in NSSI samples vary, with 65% [4] to 92% [13] in community samples, and 20 to 33% [14] and over 98% [3] in clinical samples. These variations may be partly explained by differences in assessment of psychosocial functioning. To our knowledge, In-Albon, Ruf & Schmid (2013) was the only study assessing psychosocial functioning in a moderately sized sample of 73 adolescents using clinical ratings [14]. Expert-based clinical ratings offer more beneficial validity, whereas self-ratings are often subject to misperception or cognitive biases [15, 16]. Variations in psychosocial functioning in NSSI populations may also be attributable to the population under study (adults vs. adolescents) or further risk factors such as childhood maltreatment (CM) or comorbidity. More work is needed to highlight psychosocial functioning in adolescent NSSI samples which implements clinical ratings of functioning, features larger sample sizes and sheds light on potential risk factors in this context.

While studies on NSSI and psychosocial functioning are rare, a link between childhood maltreatment (CM), i.e. experiences of sexual, physical and emotional abuse as well as physical and emotional neglect during childhood and adolescence, and functional impairments has been reliably established [17]. The often complex sequelae of CM have been shown to impact various domains of psychosocial functioning: Individuals with CM show deficits in interpersonal functioning [18], high risk of re-victimization [19, 20], higher rates of criminality and high risk of incarceration in adulthood [21]. CM has further been shown to predict school problems [22, 23] and difficulties in academic and occupational settings such as truancy or lower likelihood to attend college. Above all, however, CM is one of the most salient risk factors for mental disorders such as major depression, borderline personality disorder (BPD), or posttraumatic stress disorder (PTSD), and has been linked with maladaptive regulatory functioning and coping in behavior and neurobiology [17, 24–26]. Evidence from longitudinal data suggests that CM predicts adolescent psychological and behavioral problems above and beyond the effects of other early risk factors such as poverty or family stress [27]. Rogosch and colleagues [28] showed that CM leads to problems in affective and emotional processing that in turn lead to problems in peer relationships. These findings are supported by neurobiological evidence suggesting complex disturbances in neurodevelopmental pathways in individuals with CM [26]. CM and subsequent neurobiological alterations may also contribute to maladaptive coping patterns inherent in psychiatric disorders which in turn foster significant intra- and interpersonal problems in various domains long into adulthood [29]. In line with this, prior work has debated whether mentally ill individuals with or without CM may represent biologically distinguishable subtypes [30, 31].

Adolescents engaging in NSSI frequently report CM [9–11]. While several studies suggest a crucial role of CM in NSSI development [9, 32, 33], there is also a substantial group of individuals engaging in NSSI who do not report CM [9, 34]. It is yet unclear whether these two groups differ with respect to their levels of psychosocial functioning. As CM is associated with a significantly higher risk for mental disorders [24, 35], it is possible that CM may represent an additional risk factor that further diminishes the availability of adequate behavioral and physiological coping strategies in adolescents with NSSI, who then may present with lower psychosocial functioning.

Clinical research has suggested that CM increases mental disorder symptom severity, comorbidity and reduces treatment response in patients [36–43]. Both a higher symptom load and potentially reduced treatment benefits would put NSSI patients with CM at risk for lower psychosocial functioning compared with NSSI patients without CM. However, a potential role of mental disorder severity in differentiating psychosocial functioning in NSSI adolescents with or without CM has not been tested. Two disorders may be particularly relevant in context of NSSI, CM and psychosocial functioning: BPD and depression are most frequently diagnosed in adolescents with NSSI [44, 45] and are strongly linked with psychosocial impairment [46–48]. Moreover, both BPD [49–51] and depression [36–38, 52] are among the disorders most frequently developed after experiences of CM. CM shows stronger associations with BPD than with other diagnoses both in adolescence [49] and adulthood [53, 54], and more severe CM has been found to be associated with higher levels of BPD in NSSI adolescents specifically [55]. CM is further associated with a higher risk of developing PTSD [56–58], however, there are studies suggesting that in adolescent samples, only a low percentage (13%) of adolescents with CM meet criteria for a PTSD diagnosis [59]. Similarly, data from a large clinical sample shows that a PTSD diagnosis in adolescents with NSSI (and in part CM) is much less frequently reported than BPD or depression [45]. Still, PTSD diagnosis should be controlled for when investigating the role of BPD and depression in NSSI adolescents with or without CM.

### The present study

Considering the lack of studies examining psychosocial functioning in adolescents engaging in NSSI, our aim was to investigate psychosocial functioning in a large clinical NSSI-sample and to examine whether adolescents with or without CM may present with different levels of psychosocial functioning. In addition, we examined whether mental disorder severity may explain the difference in psychosocial functioning between those groups. We specifically focused on BPD and depression, as those disorders have been closely linked with CM but also with impairments in psychosocial functioning and are frequent in clinical samples of adolescents engaging in NSSI [45, 46, 48]. Further, we examined whether a PTSD diagnosis may explain additional variance in psychosocial functioning [58].

## Methods

### General procedure and study sample

Subjects for the present analyses were drawn from a consecutive clinical sample of adolescents presenting at the specialized German outpatient clinic for risk-taking and self-harm behavior (AtR!Sk; *Ambulanz für Risikoverhalten und Selbstschädigung*) at the Clinic for Child and Adolescent Psychiatry, Centre of Psychosocial Medicine, University of Heidelberg. AtR!Sk aims to clinically assess the engagement in risk-taking (i.e., binge-drinking, drug abuse, excessive media or internet use, promiscuity, delinquent behavior) and/or self-harm behavior (NSSI and/or suicide attempts) in adolescents aged 12–17 in order to refer them to subsequent treatment [60]. The data were collected at baseline of the ongoing AtR!Sk cohort study, which was approved by the Ethical Committee of the Medical Faculty, Heidelberg University, Germany (Study: ID S-449/2013) in accordance to the Declaration of Helsinki. Informed and written consent was provided by adolescents and their parents or other caregivers prior to inclusion in the study. Only adolescents with NSSI-disorder according to DSM-5 (≥5 acts of NSSI in the past year) and complete data were included in the present analyses. The final sample comprised *n* = 368 adolescents (*n* = 335 female, 91.03%; mean age 14.92 years, *SD* = 1.43). Based on the NSSI sample two sub-groups were generated: NSSI without CM (NSSI only, *n* = 107) and NSSI with CM (NSSI+CM, *n* = 261). The two groups did not differ on age and sex distribution, but significantly differed on school type, number of BPD and depression score (further details see results).

### Clinical assessment

#### Semi-structured interviews

To assess BPD symptoms dimensionally, the German version [61] of the *Structured Clinical Interview for DSM-IV-Axis II* (SCID-II; [62]) was used. The SCID-II is widely known to be suitable for the use in adolescents [63].

The German version of the *Self-Injurious Thoughts and Behavior Interview* (SITBI-G; [64]) was used to assess NSSI and suicidal behavior. The SITBI-G shows excellent reliability and validity in adolescent samples [64].

The German version of the *Mini-International Neuropsychiatric Interview for Children and Adolescents* (M.I.N.I-KID 6.0; [65]) was conducted to assess current psychiatric diagnoses. The M.I.N.I.-KID is a brief structured clinician rated diagnostic interview for DSM-IV and ICD-10 psychiatric disorders for children and adolescents aged 6–19 years and has demonstrated good reliability and validity [66].

All interviews were carried out by experienced clinicians in the field of adolescent BPD. Study therapists were clinical psychologists (Master in clinical psychology) who were in training to become licensed psychotherapists, and in addition, training to become DBT-A therapists. They already completed their intermediate exams after 1.5 years. Within AtR!Sk, assessors underwent regular reliability checks. To check for inter-rater reliability, audiotaped interviews of each clinician, consisting of the M.I.N.I.-Kid, the SCID II (borderline, avoidant, dependent and antisocial personality disorder), as well as the SITBI-G were recorded. Interviews were assessed by independent second raters blind to the first raters’ scores and diagnoses. Concerning the SITBI-G, very good to perfect agreements were found within the inter-rater reliability checks (κs = 0.77–1.00). Regarding the SCID II, diagnostic agreement for full-threshold BPD was at 93.6%.

#### Self-report measures

Based on the *Children’s Depression Inventory* [67] the German *Depressions-Inventar für Kinder und Jugendliche* (DIKJ; [68]) was used as a self-report measure to assess depression symptoms in adolescents in the last 2 weeks based on diagnostic criteria for depressive disorder. The DIKJ demonstrated good validity and reliability in clinical samples [68] and α = 0.87 in the current sample. CM was evaluated with the German version [69] of the *Childhood Experience of Care and Abuse Questionnaire* (CECA.Q; [70]). The CECA. Q assesses various types of early traumatic childhood experiences retrospectively. It describes experiences of parental antipathy, neglect, and abuse before the age of 17 years. The German version of the CECA. Q showed good internal consistency for each subscale (Cronbach’s α from 0.88 to 0.92) and retest reliability (Cohen’s k from 0.78 to 0.93) [69]. For the present analyses a dichotomous variable (CM: yes/no) was generated.

#### Clinical rating of psychosocial functioning

Psychological, social and occupational functioning were assessed at the end of the diagnostic interview with the *Global Assessment of Functioning Scale* (GAF), a numerical rating scale from 1 to 100 usually reported on the axis V of the multiaxial diagnostic system in DSM-IV [71]. The higher the score, the better the current level of psychosocial functioning. A GAF score between 41 and 50 indicates serious symptoms (e.g., suicidal ideation, severe obsessional rituals, frequent shoplifting) or any serious impairment in social, occupational, or school functioning (e.g., no friends, unable to keep a job, cannot work). GAF scores were assessed by experienced clinicians in the field of adolescent NSSI.

### Statistical analyses

To test differences in GAF mean scores in the NSSI only and NSSI+CM groups, the two groups were compared with analysis of variance (ANOVA). Because of significant differences in sex distribution and school type these variables were added as covariates in the adjusted model (ANCOVA). To analyze whether BPD and depression severity may explain the differences in GAF scores between the two groups, first descriptive pairwise correlations between all dimensional variables (number of BPD symptoms assessed via SCID-II, depression assessed via DIKJ and GAF) and age were calculated to test for associations and confounding effects between these variables of interest. To test the effect of sex on BPD, depression and GAF point biserial correlation effect sizes were calculated. Reported *p*-values stem from t-tests, respectively. Šidák-correction was used for correction of multiple comparisons. Mediational hypotheses were tested by employing structural equation modelling (SEM) with maximum likelihood (ML) estimation. The model tested whether CM predicted a lower level of psychosocial functioning (lower GAF) directly and indirectly via BPD features and depression symptoms. The full model included all paths between the variables CM, BPD, depression and GAF. To adjust for PTSD we integrated categorial PTSD diagnosis in the first model. Goodness of fit (GOF) was assessed using the comparative fit index (CFI), the Tucker Lewis Index (TLI) and the root mean square error of approximation (RMSEA). RMSEA ≥0 and ≤ 0.05, CFI ≥ 0.97 and TLI ≥ 0.97 are indicators of a good model fit [72]. Effects were tested by normal-based bootstrapped confidence intervals (CIs). All analyses were performed using Stata 15.1 (Version 15.1; StataCorp LP, College Station, TX, US) and alpha set to .05.

## Results

### Descriptive analyses

#### Total sample

The total sample consisted of *n* = 368 adolescents meeting DSM-5 criteria for NSSI. Psychiatric diagnoses within the AtR!Sk cohort were classified based on the International classification of diseases according to WHO (ICD-10; [73]). The most frequently met comorbid disorders were affective disorders (F3) (*n* = 249, 67.66%, and of those *n* = 231, 62.77% with a current depression diagnosis), and personality disorders (F6) (*n* = 181, 49.18%, out of whom *n* = 137, 37.23% met criteria for full-threshold BPD), followed by neurotic, stress-related, and somatoform disorders (F4) (*n* = 158, 42.93%, of those *n* = 24, 6.52% with a PTSD diagnosis), and behavioral and emotional disorders with onset usually occurring in childhood and adolescence (F9) (*n* = 102, 27.72%). A mean GAF score of 47.89 (*SD* = 10.94) was reported which indicates serious symptoms. The mean number of BPD criteria in the total sample was 3.75 (*SD* = 2.19). The mean DIKJ score was 30.94 (*SD* = 8.72), corresponding to a percentile rank of 98.7.

#### Subgroups

To test *differences in GAF scores,* the total NSSI sample was split in two subgroups: NSSI without CM (NSSI only) (*n* = 107) and NSSI+CM (*n* = 261). Of those with CM, *n* = 93 (25.62% of the total sample) reported sexual abuse, *n* = 211 (58.29%) experienced antipathy from their caregivers, *n* = 154 (42.66%) neglect and *n* = 96 (26.30%) physical abuse. Detailed sociodemographic and clinical characteristics between the groups are reported in Table 1. The two groups did not differ regarding their sex (χ^2^_(1)_ = 3.13, *p* = 0.077) and age (F_(1, 366)_ = 0.76, *p* = 0.384), but significantly differed in school type (χ^2^_(3)_ = 14.16, *p* = 0.003), number of BPD criteria met (F_(1, 366)_ = 21.68, *p* <  0.001) and depression severity in the DIKJ (F_(1, 366)_ = 21.39, *p* <  0.001). The NSSI+CM group endorsed more BPD criteria (4.08 vs. 2.94) and greater depression severity (32.25 vs. 27.75).

**Table 1:** Sociodemographic and main outcome variables within the NSSI sample

	NSSI only (***n*** = 107)		NSSI + ACEs (***n*** = 261)		***p***
	*M*	*SD*	*M*	*SD*	
Age	14.82	1.55	14.97	1.38	*0.401*
BPD criteria	2.94	2.05	4.08	2.17	*< 0.001*
DIKJ	27.75	8.47	32.25	8.49	*< 0.001*
GAF	50.93	12.13	46.64	10.18	*< 0.001*
	***N***	***%***	***N***	***%***	
Females	93	86.92	242	92.72	*0.077*
School type^a^					*0.003*
Hauptschule	7	6.54	31	11.92	
Realschule	28	26.17	99	37.69	
Gymnasium	57	53.27	85	32.69	
Other	15	14.02	46	17.70	
Household composition					*0.181*
With biological mother	92	86.79	192	75.89	
With other mother figure	4	3.78	11	4.35	
With no mother figure	10	9.43	50	19.76	
With biological father	62	60.78	111	46.25	
With other father figure	11	10.79	39	16.25	
With no father figure	29	28.43	90	37.50	
Residential youth service	5	4.67	34	13.03	

^a^Hauptschule: 9 years of elementary school; Realschule: 6 years of school after 4 years of elementary school, terminating with a secondary school level-I certificate; Gymnasium: 8 years of school after 4 years of elementary school, terminating with the general qualification for university entrance. Percentage scores take account of missing values

### Group differences

**Fig. 1:**
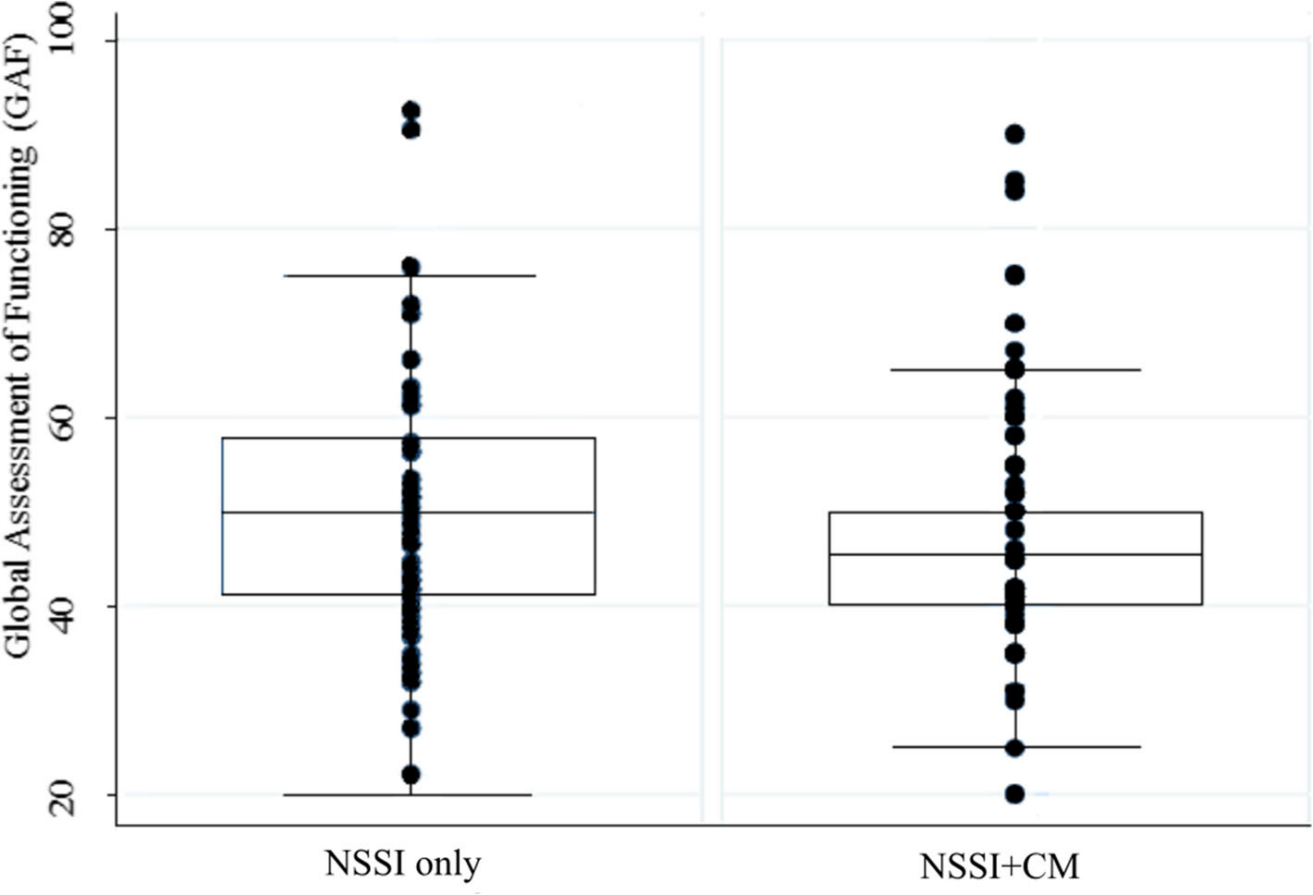
Group differences between adolescents with NSSI only and adolescents with NSSI and CM on GAF

The mean GAF score in the NSSI only group was 50.92 (*SD* = 12.13) vs. 46.64 (*SD* = 10.18) in the NSSI+CM group. Group differences on GAF based on single data points are illustrated in Fig. 1. ANCOVA yielded significant differences between the groups on GAF with school type not adding further information as covariate (F_(4)_ = 4.48, *p* = 0.002).

### Structural equation model

Before computing SEM, basic associations between all variables of interest (CM, BPD, depression, GAF) and possible confounding variables (sex, age) were tested. Pairwise correlation analysis yielded significant correlation between BPD, depression and GAF. Age was associated with number of BPD criteria and GAF but not with depression. Point biserial correlation showed significant effects of CM on all other variables of interest. Sex was associated with depression but not with BPD and GAF. Šidák-corrected correlation coefficients, point biserial effect sizes and respective *p*-values are reported in Table 2.

**Table 2:** Pairwise correlation (r) matrix and point biserial correlation (r_pb_) effect sizes

Variables	BPD	DIKJ	GAF
Pairwise			
Age	0.282***	−0.006	− 0.139*
BPD	–	0.242***	−0.353***
DIKJ		–	−0.293***
GAF			–
Point-biserial			
CM	.236***	.235***	-.178***
Sex	0.073	0.127*	−0.013

CM=childhood maltreatment, *DIKJ *= Depressionsinventar für Kinder- und Jugendliche (depression inventory for children and adolescents), *BPD* = borderline personality disorder, *GAF* = global assessment of functioning; ***=*p* < 0.001, *=*p* < 0.05

Based on the NSSI sample the first model (2.1) included all direct paths between the variables CM, BPD, depression and GAF. To account for confounding effects, covariance between BPD and depression was added to the model and additional paths from sex ➔ depression and age ➔ BPD/GAF. An additional model integrating PTSD diagnosis as a third potential mediator neither revealed a significant effect for PTSD nor did it explain additional variance in the data; thus, PTSD was renounced in the final model.

**Fig. 2:**
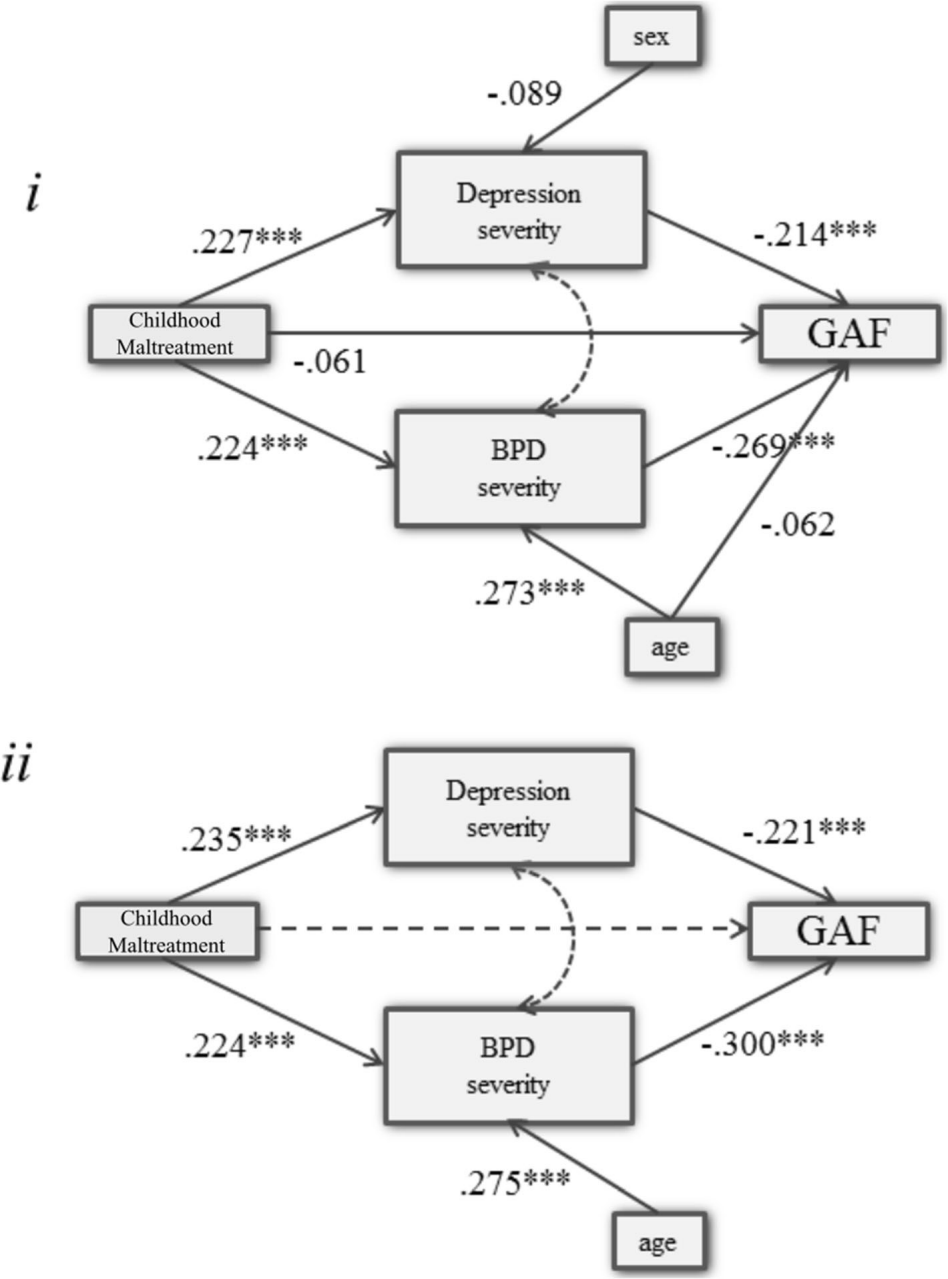
Structural equation models for the pathways from CM to GAF. Normal-based bootstrapped parameter MLM estimates are reported as standardized *β*-coefficients. The straight dashed line represents the indirect effect. The curved dashed lines represent covariance; *** = *p* < 0.001

CM directly predicted BPD and depression severity. BPD and depression severity further directly predicted GAF. The direct pathway from CM to GAF did not reach significance in this model. Sex had no influence on depression and age was only associated with BPD but not with GAF. The model provided a good fit (CFI = 0.998, TLI = 0.991, RMSEA = 0.018). In the next step, non-significant paths were eliminated for parsimony (model 2.2). All the ML-based parameter estimates were statistically significant (*p* <  0.001). CM predicted GAF only indirectly via BPD symptoms and depression severity. Model 2.2 yielded better GOF than model i (CFI = 0.999, TLI = 0.997, RMSEA < 0.012). Standardized coefficients and CIs are reported in Table 3. The computed SEMs are illustrated in Fig. 2*.*

**Table 3:** Bootstrapped standardized β-coefficients and confidence intervals (CIs) for the structural equation models

	β	SD	***p***	95% CI	
***2.1***					
BPD					
CM	0.224	0.045	*< 0.001*	0.136	0.312
age	0.273	0.042	*< 0.001*	0.190	0.355
Depression					
CM	0.227	0.043	*< 0.001*	0.143	0.311
sex	−0.089	0.465	*0.056*	−0.180	0.002
GAF					
BPD	−0.269	0.050	*< 0.001*	−0.366	−0.172
Depression	−0.214	0.522	*< 0.001*	−0.316	−0.111
CM	−0.061	0.059	*0.297*	−0.176	0.054
age	−0.062	0.043	*0.148*	−0.145	0.022
***2.2***					
BPD					
CM	0.224	0.055	*< 0.001*	0.116	0.332
age	0.275	0.038	*< 0.001*	0.200	0.349
Depression					
CM	0.235	0.058	*< 0.001*	0.122	0.348
GAF					
BPD	−0.300	0.042	*< 0.001*	−0.383	−0.216
Depression	−0.221	0.054	*< 0.001*	−0.327	−0.114

### Model with categorical diagnoses

In order to validate the results from SEM, linear regression analyses with CM as predictor for GAF were calculated in the total sample. CM significantly predicted GAF in the regression model (F_(1.338)_ = 9.54, *p* = .002). However, when categorical BPD and current depression diagnoses were taken into the regression model as covariates, CM as predictor for GAF had no significant value (β = − 1.94, t = − 1.51, *p* = .133) compared to BPD (β = − 6.87, t = − 5.75, *p* < .001) and depression (β = − 4.11, t = − 3.50, *p* = .001), with the model itself yielding a significant result (F_(3.336)_ = 17.62, *p* < .001). Adding sex and school type as covariates did not significantly add further explanation of variance.

## Discussion

Our results demonstrate that adolescents with NSSI and CM have significantly lower levels of psychosocial functioning (lower GAF scores) than adolescents engaging in NSSI who do not report CM. They also suggest that CM is indirectly linked with lower psychosocial functioning such that severity of BPD and depression (but not PTSD) explains the differences in psychosocial functioning in NSSI adolescents with vs. without CM. Thus, our findings are in line with prior work suggesting that individuals with mental disorder and additional CM differ from individuals without CM in their level of functioning [30, 31], and that these differences can be explained by different symptom profiles and more severe mental disorder [38–40, 43, 51, 74]. They contribute to the ongoing debate as to whether mentally ill individuals presenting with CM and those with the same mental disorder but without CM may represent specific ecophenotypes [39] but may rather suggest that CM is associated with higher illness severity of specific disorders.

Our study suggests that patients who report both NSSI and CM present with higher levels of BPD symptoms and depression severity and lower GAF scores. CM are uncontrollable and painful events that undermine the child’s development of functional coping patterns, that are associated with cognitive and affective processing biases like hyperarousal or biased attention towards threat [75] and ineffective emotion regulation strategies already present in NSSI [76, 77].

Besides its contribution to the accumulation of dysfunctional emotion regulation strategies, CM contributes to a range of further problems such as re-traumatization, adverse environments and bullying experiences [78–81] that can further fuel emotional and behavioral problems in already vulnerable adolescents, contributing to more severe conditions that are associated with significantly poorer functioning. Thus, CM may serve as an amplifying factor for higher levels of distress and lower levels of functioning in NSSI adolescents that are already at risk.

Our findings indicate that severity of BPD and depression is independently linked with psychosocial functioning and that both disorders equally explain variance in psychosocial functioning. Both disorders are strongly linked with poor emotion regulation [82, 83] and associated with impaired psychosocial functioning [46–48]. Future studies should examine whether emotion dysregulation may be a common factor explaining the relevance of both disorders with regard to psychosocial functioning. A PTSD diagnosis did not explain further variance in the model. Prior work has suggested that a relatively low percentage of adolescents who report CM also meet criteria for PTSD, whereas BPD and depression seem to be more frequently diagnosed in adolescents with NSSI [45, 59] In line with this, in the current sample, 62.8% had a diagnosis of depression and 37.0% had a diagnosis of BPD, whereas only 6.52% of patients had a diagnosis of PTSD. This low prevalence of PTSD in our and other samples may be due to the nature of traumatic experiences assessed: Our assessment using the CECA. Q was mainly focused on chronic and enduring childhood adversity within the family environment (Complex or Type-II trauma), which has been shown to be specifically associated with depression and personality disorder development and may not be as strongly associated with PTSD [24, 35, 38, 49, 52]. The discussion around diagnosing a trauma-related disorder in patients with complex CM is an issue of intense scientific debate [84]. After the notion of a separate trauma-related disorder for patients suffering from complex trauma was rejected in the DSM, the emergence of the diagnosis of “complex PTSD” in the new ICD-11 will certainly give new impulses. However, it would have been very interesting to be able to examine the effects of complex PTSD in our sample. Unfortunately, we did not implement measures to assess of this specific symptom profile in order to be able to include the respective information in our models.

### Limitations

The cross-sectional nature of our study does not permit causal interpretations. Future studies would benefit from longitudinal designs that allowed to draw conclusions about the effects of CM on the development and severity of mental disorders and psychosocial functioning over time. Further, we implemented only one index of psychosocial functioning, the GAF. There have been some concerns regarding the GAF’s reliability and validity [75, 85]. When the present study was designed in 2013, the GAF was chosen based on its widespread use as a clinical rating tool within the DSM-IV. Future studies should implement measures with more satisfying psychometric properties. Adolescents’ CM assessment was based on retrospective self-reports that could reflect bias of autobiographical memory rather than the influence of the event per se. A recent meta-analysis pointed out that there is poor agreement between prospective and retrospective measures of CM, and that those two types of measures identify largely different groups of individuals [86]. At the same time, studies report that prospectively and retrospectively assessed CM seems to be linked with similarly elevated risk of mental disorder, suggesting a robust effect of CM in both types of measures [24, 87]. We further did not assess at what age exactly maltreatment experiences occurred. Prior work has indicated that individuals with experiences of maltreatment before the age of 12 are at higher risk for depression, while those experiencing maltreatment after 12 years are at higher risk for PTSD [88]. Further, childhood maltreatment between three and five years of age is associated with more adverse mental health outcomes in adulthood than maltreatment earlier or later in childhood [74]. These findings highlight the importance of sensitive time periods and their prognostic influence on mental disorder that should be implemented in future research designs. Further, our analyses were based on a dichotomous (yes/no) CM variable that does not specify distinct types and severity of maltreatment events. Research shows that different forms of childhood maltreatment predict unique variance in emotion regulation strategies [89] that could specifically be associated with mental disorder. Yet, the aim of the study was to investigate a global effect of CM without a focus on a potential dose-response-relationship. 

It should be mentioned that the sample was predominantly female. Further research should address if the reported associations may vary depending on sex. Unfortunately, the small number of male adolescents in our sample did not permit these additional analyses. Lastly, our sample consisted of individuals with NSSI disorder, and comorbidity was rather high. We based our analyses on the most frequently reported disorders, BPD and depression, and further included PTSD, however, other psychiatric disorders such as anxiety might have influenced our findings. Further research could examine whether the reported findings can be replicated in samples with a different distribution of comorbid disorders.

## Conclusion

To the best of our knowledge, this is the first study to investigate the association of CM and psychosocial functioning in a large clinical sample of adolescents with NSSI which further implemented clinical expert ratings of psychosocial functioning. Our results implicate that clinicians need to be aware that additional CM may render adolescents with NSSI more vulnerable for more severe disorders and thus lower psychosocial functioning. Specifically, BPD and depression should be targeted in order to support NSSI patients and increase psychosocial functioning. Specialized treatments for adolescents such as dialectical-behavioral therapy (DBT-A) or mentalization-based therapy (MBT-A) are reported to be effective in reducing symptoms of emotion dysregulation, depression and interpersonal problems in these clinical populations [90, 91]. Furthermore, promising effects of a short-term intervention on NSSI with similar effect sizes have recently been published [92]. BPD and depression are treatable, and therapeutic interventions may buffer adverse effects of CM in adolescents with NSSI.

## Data Availability

The datasets used and/or analyzed during the current study are available from the corresponding author on reasonable request.
